# *Toxoplasma gondii *down modulates cadherin expression in skeletal muscle cells inhibiting myogenesis

**DOI:** 10.1186/1471-2180-11-110

**Published:** 2011-05-18

**Authors:** Alessandra F Gomes, Erick V Guimarães, Laís Carvalho, José R Correa, Leila Mendonça-Lima, Helene S Barbosa

**Affiliations:** 1 Laboratório de Biologia Estrutural, Instituto Oswaldo Cruz, Fundação Oswaldo Cruz, (Av. Brasil 4365), Rio de Janeiro (21040-361), Brazil; 2 Laboratório Cultura de Células, Instituto de Biologia, Universidade do Estado do Rio de Janeiro, (Av. Prof. Manoel de Abreu 444), Rio de Janeiro (20550-170), Brazil; 3 Laboratório de Genômica Funcional e Bioinformática, Instituto Oswaldo Cruz, Fundação Oswaldo Cruz, (Av. Brasil 4365), Rio de Janeiro (21040-361), Brazil

**Keywords:** *Toxoplasma gondii*, myogenesis, cadherin, skeletal muscle cells, *T. gondii*-host cell interaction

## Abstract

**Background:**

*Toxoplasma gondii *belongs to a large and diverse group of obligate intracellular parasitic protozoa. Primary culture of mice skeletal muscle cells (SkMC) was employed as a model for experimental toxoplasmosis studies. The myogenesis of SkMC was reproduced *in vitro *and the ability of *T. gondii *tachyzoite forms to infect myoblasts and myotubes and its influence on SkMC myogenesis were analyzed.

**Results:**

In this study we show that, after 24 h of interaction, myoblasts (61%) were more infected with *T. gondii *than myotubes (38%) and inhibition of myogenesis was about 75%. The role of adhesion molecules such as cadherin in this event was investigated. First, we demonstrate that cadherin localization was restricted to the contact areas between myocytes/myocytes and myocytes/myotubes during the myogenesis process. Immunofluorescence and immunoblotting analysis of parasite-host cell interaction showed a 54% reduction in cadherin expression at 24 h of infection. Concomitantly, a reduction in M-cadherin mRNA levels was observed after 3 and 24 h of *T. gondii-*host cell interaction.

**Conclusions:**

These data suggest that *T. gondii *is able to down regulate M-cadherin expression, leading to molecular modifications in the host cell surface that interfere with membrane fusion and consequently affect the myogenesis process.

## Background

*Toxoplasma gondii *is an obligatory intracellular parasite and an important human pathogen. Humans acquire toxoplasmosis due to oocyst seeding from cats, consumption of raw or undercooked meat or vertical transmission to the fetus during pregnancy. Studies of environmental factors in several communities indicated an important role for cultural and eating habits on this infection transmission [1]. During natural vertical infections, *Toxoplasma *initially crosses the intestinal epithelium of the mother, disseminates into the deep tissues and traverses the placenta, the blood-brain and the blood-retina barriers [2]. In both immunocompromised and immunocompetent individuals, *Toxoplasma *infection can cause a severe ocular pathology [3,4]. These parasites are able to invade and rapidly replicate in any nucleated host cell and may develop cysts, predominantly in neural and muscular tissues, initiating the chronic infection stage.

Until now little attention has been given to skeletal muscle as a model in experimental toxoplasmosis studies [5-9], though skeletal muscle is one of the main sites for the occurrence of cystogenesis [10].

It is established that toxoplasmosis can cause myositis either by recent infection or by infection reactivation, causing muscle injury and release of parasites in the bloodstream [11,12]. The involvement of muscular tissue in the chronic stage of toxoplasmosis is a significant clinical aspect for immunodeficient individuals infected with the HIV virus, and can be employed in biopsies for diagnosis, as proposed by [13]. In addition, one case of polymyositis in an immunocompetent patient diagnosed with acquired toxoplasmosis has been reported [14]. The interaction of *T. gondii *and primary cultures of skeletal muscle cells has been exploited by our group. This model reproduces important characteristics of the *in vivo *infection and also allows *in vitro *cystogenesis analysis [5-9,15-17]. The dynamics of SkMC cultures obtained from mouse embryos allows the investigation of each myogenesis stage [18,19].

The adhesive contact regulation between cells underlies many morphogenetic processes during the development of new tissues and the controlled growth and turnover of adult tissues. The cell-cell physical interaction that occurs during myogenesis is carried out by cellular adhesion molecules. However, cadherins, comprising a family of adhesion molecules, are particularly important to the dynamic regulation of adherent junctions, which are associated with diverse morphogenetic processes [20]. Several intracellular pathogens able to modulate adhesion molecules on this junction during the infectious process may cause tissue pathogenesis [21-25]. During the myogenesis process, M-cadherins (M for muscle) are involved in the initial cell-cell recognition, allowing initiation of myoblast fusion to form multinucleated myotubes [26,27], as demonstrated by the RNA interference method [28].

In the present study, we examined: (i) *T. gondii *tachyzoite capacity to infect SkMC (myoblasts and myotubes); (ii) the influence of *T. gondii *infection on myogenesis process; (iii) the parasite's impact on SkMC M-cadherin expression and, (iv) its correlation with myogenesis process.

## Methods

All procedures were carried out in accordance with the guidelines established by the Colégio Brasileiro de Experimentação Animal (COBEA), by Fundação Oswaldo Cruz-Fiocruz, Committee of Ethics for the Use of Animals (license CEUA LW 10/10) and by Guidelines on the Cared and Use of Animals for Experimental Purposes and Infectious Agents (NACLAR).

### Primary culture of skeletal muscle cells

SkMC cultures were obtained from thigh muscles of 18-day-old mouse embryos. The tissues were minced and incubated for 7 min with 0.05% trypsin and 0.01% versene diluted in phosphate-buffered saline pH 7.2 (PBS). After 5-7 dissociation cycles, the enzymatic digestion was interrupted by addition of 10% fetal bovine serum at 4°C. The suspension was centrifuged at 650 g for 7 min, resuspended in Dulbecco's modified Eagle medium (DMEM) supplemented with 10% horse serum, 5% fetal bovine serum, 2% chick embryo extract, 1 mM *L*-glutamine, 1,000 U/mL penicillin, 50 μg/mL streptomycin and then incubated for 30 min at 37°C in a 5% CO_2 _atmosphere. After incubation, the culture flask was gently shaken to release non-attached cells and the supernatant enriched with myoblasts was seeded in 0.02% gelatin-treated 24-well culture plates for the fluorescence assays. The cultures were maintained at 37°C up to 2-5 days to obtain the muscle fibers and fresh culture medium was added every two days.

### Parasites

Tachyzoites of *T. gondii*, RH strain, were maintained in Swiss mice by serial intraperitoneal inoculation of 10^5 ^parasites. After 48-72 h the parasites were harvested in PBS and centrifuged (200 g for 7-10 min) at room temperature in order to discard blood cells and cellular debris. The supernatant was collected and then centrifuged again at 1000 g for 10 min. The final pellet was resuspended in DMEM and used in the interaction assays.

### *T. gondii *infection during skeletal muscle cell myogenesis

Aiming to verify the infectivity of *T. gondii *in myoblasts and myotubes, we developed the following protocol: 2-day-old cultures were infected with tachyzoite forms (1:1 parasite-host cell ratio) and, after 24 h of interaction, the total number of infected myoblasts and myotubes was quantified independent of the number of internalized parasites.

For evaluation of the potential interference of *T. gondii *in myotube formation, after the initial seeding, cultures were maintained for 48 h in medium without calcium, in order to not stimulate myoblast fusion. After this time, the cultures, enriched in myoblasts, were infected for 24 h. Cell fusion in the presence or absence of *T. gondii *was determined by morphological analysis of myoblast alignment and the observation of the percentage of multinucleated cells.

The quantitative analysis was based on 3 independent experiments performed in duplicate with at least 200 cells in each coverslip.

### Fluorescence analysis of actin microfilaments

SkMC 2-day-old cultures were allowed to interact with tachyzoites (1:1 parasite: host cell ratio) for 24 and 48 h at 37°C. Non-infected and infected SkMC were fixed for 5 min at room temperature in 4% paraformaldehyde (PFA) diluted in PBS. After fixation, the cultures were washed 3 times (10 min each) in the same buffer. Then, the cultures were incubated for 1 h at 37°C with 4 μg/ml phalloidin-rhodamine diluted in PBS. Thereafter, the cultures were washed 3 times (10 min each) in PBS, incubated for 5 min in 0.1 μg/mL DAPI (4',6-diamidino-2-phenylindole, Sigma Chemical Co.), a DNA stain that enables the visualization of host and parasite nuclei, and washed again in PBS. The coverslips were mounted on slides with a solution of 2.5% DABCO (1,4-diazabicyclo-[2]-octane-triethylenediamine antifading, Sigma Chemical Co.) in PBS containing 50% glycerol, pH 7.2. The samples were examined in a confocal laser scanning microscope (CLSM Axiovert 510, META, Zeiss, Germany) from the Confocal Microscopy Plataform/PDTIS/Fiocruz, using a 543 helium laser (LP560 filter) and 405 Diiod laser (LP 420 filter).

### Immunofluorescence analysis of total cadherin protein distribution in SkMC myogenesis during infection with *T. gondii*

Immunofluorescence assays were performed using specific monoclonal antibodies for pan-cadherin (Sigma Chemical Co. C3678). Briefly, tachyzoite forms were allowed to interact with 2-day-old SkMC in the ratio of 1:1. After 3, 12, and 24 h of interaction, the cultures were fixed for 5 min at room temperature in 4% paraformaldehyde diluted in PBS and then washed 3 times (10 min each) with PBS. The cultures were incubated for 1 h at room temperature in blocking solution containing 4% bovine serum albumin (BSA) and 0.5% Triton X100 (Sigma Chemical Co.) in PBS, followed by incubation overnight at 37°C with anti-pan cadherin antibody diluted 1:200 in PBS/BSA. The cultures were washed 3 times (10 min each) in PBS/BSA and incubated for 1 h at 37°C with Alexa Fluor 488, goat anti-rabbit IgG (Invitrogen, Molecular Probes) diluted 1:1000 in PBS/BSA. Coverslips were subsequently washed 3 times (10 min each) in PBS, incubated for 10 min in 0.1 μg/mL DAPI and washed again in PBS. Coverslips were mounted on slides and examined by confocal microscopy as described above. Controls were performed by omission of the primary antibody.

### Western blot analysis

For western blot analysis of total cadherin pool, the proteins were extracted from the following samples: (a) 2-day-old SkMC to observe the protein synthesis pattern before infection; (b) 3-day-old SkMC (uninfected control) and, (c) SkMC infected with *T. gondii *tachyzoites (1:1 parasite:host-cell ratio), 24 h after infection (to study the possible impact of *T. gondii *infection in cadherin expression). Cadherin expression by *T. gondii *protozoan alone was also verified by western blot assays.

Cells were washed with PBS and maintained in ice for protein extraction. Briefly, cells were collected in approximately 600uL of lysis buffer (50 mM Tris-Cl pH 8, 150 mM NaCl, 100 ug/mL PMSF, 1 mg/mL pepstatine. 1 mg/mL aprotinine, 10 mg/mL leupeptine in 1% Triton X-100, 0.4 mg/mL EGTA). Cell debris were removed by centrifugation, proteins in the cleared supernatant precipitated with cold acetone and resuspended in 8 M ureum/2% CHAPS. Total protein concentration was determined with the RC-DC kit (BioRad) prior to separation in 10% SDS-PAGE gels. Proteins were electro-transferred to Hybond C membranes (GE Healthcare) with a Trans-Blot apparatus (BioRad), visualized by reversible staining with MemCode (Pierce) and the images captured in a GS-800 scanning densitometer (BioRad). Primary anti-Pan-cadherin mouse antibody (Sigma Chemical Co. C-1821) was used in a 1:2,000 dilution and bound antibodies were revealed using a peroxidase-coupled anti-mouse IgG antibody (Pierce 31430, 1:5,000 dilution). Blots were visualized with the SuperSignal West Pico chemiluminescence substrate (Pierce, 34080) and images captured as described above. For quantitative analysis, western blot signals were normalized against total proteins detected per lane in the corresponding MemCode stained membrane using the QuantityOne software (BioRad).

### RNA extraction and reverse transcription-PCR (RT-PCR)

Total RNA was extracted from SkMC culture samples harvested at three different time points during the *T. gondii *infection assay (3 h, 12 h and 24 h). For this purpose, 10^6 ^cells were harvested and washed three times in PBS and the pellet used for RNA extraction with the RNeasy kit (Qiagen California, CA, USA - 74104) according to the manufacture's recommendations. Reverse transcription was carried using 2 μg of each RNA sample and the Mix reagents acquired from BioRad (California, USA - 170-8897), following the manufacture's instructions. For cDNA amplification, gene-specific primers targeted to M-Cadherin [29] and GAPDH (glyceraldehyde 3-phosphate dehydrogenase) were used. PCR was carried out in a final volume of 10 μL, with 1 μL target cDNA, 5 pmol of each primer, 200 μM each desoxyribonucleotide triphosphate (dNTP) (Promega, Wisconsin, USA), 0.8 units TaqDNA polymerase (Cenbiot, Rio Grande do Sul, Brazil) in a buffer containing 10 mM Tris-HCl, pH 8.5, 50 mM KCl, 1.5 mM MgCl_2 _as previously described [30]. PCR analysis considered the gene expression of infected and uninfected host cells in relation to the internal control, GAPDH, as previously reported [31-35]. The samples were amplified for 30 cycles (denaturation at 94°C for 60 sec, annealing at 56°C or 54°C for M-Cadherin and GAPDH, respectively, and extension at 72°C for 60 sec). PCR products were visualized on 8% silver stained polyacrylamide gels. Gel images were acquired (Epson Perfection 4180 Photo, California, USA).

### Statistical analysis

Densitometric analysis was performed using the Image J software (NIH) or Quantity One (BioRad, for western blot quantification). Student's *t *-test was used to determine the significance of differences between means in Western blot, RT-PCR and quantitative assays. A *p *value ≤ 0.05 was considered significant.

## Results

### *T. gondii *infectivity of SkMC

**Figure 1:**
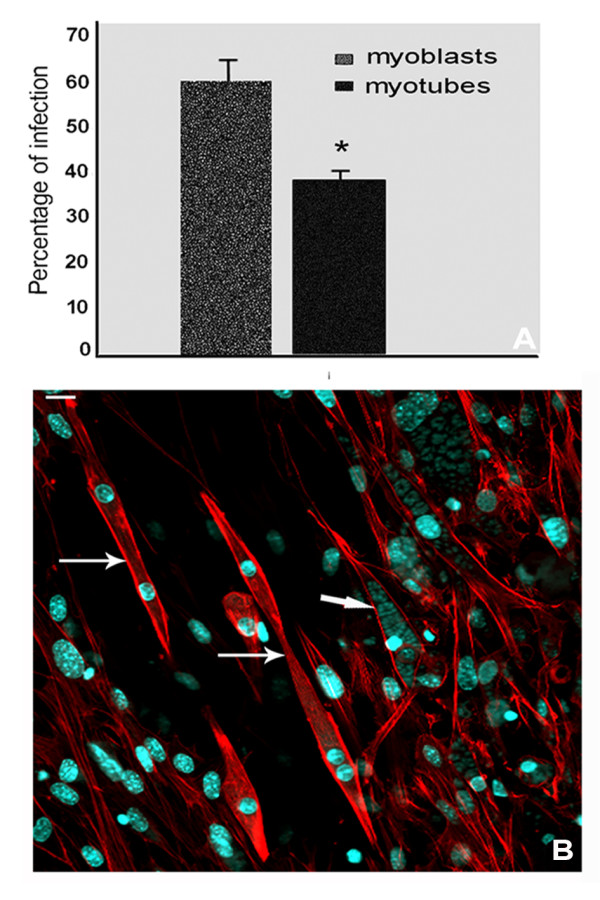
**Percentage of *T. gondii *infected SkMC after 24 h of interaction**. (A) Percentage of myoblasts (61%) and myotubes (38%) infected with *T. gondii *after 24 h of interaction. Student's T-test (*) p ≤ 0.05. (B) Details of SkMC cultures profile observed by fluorescence microscopy with phaloidin-TRITC labeling showing actin filaments in red; nuclei of the cells and the parasites labeled with DAPI, in blue. Infected cultures present myoblasts containing several parasites (thick arrow) and young myotubes with 2 nuclei without parasites (thin arrows). Bars, 20 μm

Only the number of infected myoblasts and myotubes was evaluated, independently of the number of parasites internalized. The total number of infected cells (harboring at least one internalized parasite), after 24 h of SkMC - parasite interaction, represented 61% of myoblasts and 38% of myotubes. These data indicate that myotubes were 1.6-fold less infected than myoblasts (Figure 1A). Figure 1B shows young and mature uninfected myotubes surrounded by several heavily infected myoblasts after 48 h of interaction.

### Effect of *T. gondii *infection on SkMC myogenesis

**Figure 2:**
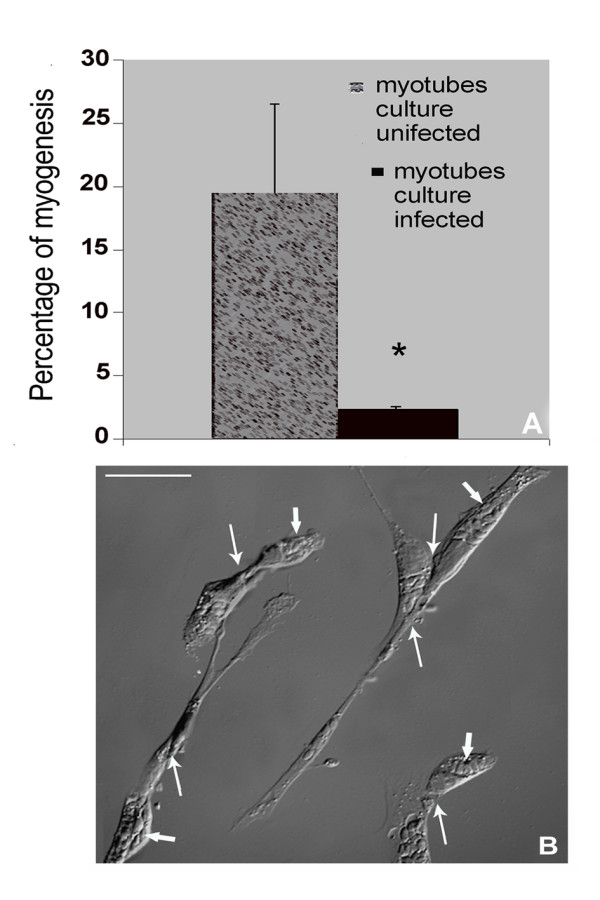
**Quantitative analysis of myotube formation percentage during myogenesis in *T. gondii *infected cultures**. (A) In uninfected cultures, after 3 days, the percentage of myotubes was 19.5% while in infected cultures, after 24 h of interaction, this percentage decreased to 2.5%. Note the 75% reduction in the formation of myotubes in infected cultures. Student's T-test (*) p = 0.0025. (B) Differential interference contrast (DIC) image showing influence of the infection by *T. gondii *(24 h of interaction) on SkMC myogenesis. Parasite (thick arrows) and unfused myocytes (thin arrows).

We also analysed the influence of *T. gondii *infection on SkMC myogenesis. Even at low parasite-host cell ratios (1:1), after 24 h of interaction, the infection percentage was 43% ± 0.06. In uninfected 3-day-old cultures the myotube percentage was 19.5% of the number of total cells. In contrast, infected 3-day-old cultures, after 24 h of infection, showed only 2.5% of multinuclear cells, representing an inhibition of 75% (p ≤ 0.05) in myotube formation (Figure 2A). Figure 2B shows that infected myoblasts kept their alignment capacity. Additionally, infected cultures, after 48 h, presented unaltered fusion of non-parasitized myoblasts. The myogenesis course in this case was maintained as demonstrated by myotube existence (Figure 1B).

### Detection of cadherin protein in SkMC during infection with *T. gondii *by immunofluorescence analysis

**Figure 3:**
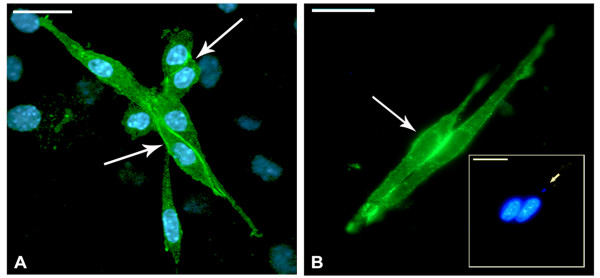
**Cadherin localization in primary SkMC cultures**. Indirect immunofluorescence assays showing: (A) 2-day-old myoblasts under multiplication and differentiation. Cadherin (in green) is strongly marked in every cell with high concentrations in edges near the membrane and points of cell-cell contact (arrows). (B) apparently, the existence of a single newly internalized parasite (inset) did not lead to any change in the profile of cadherin expression and distribution in host cells (arrow). Nuclei of cells and parasites are labeled with DAPI, in blue. Bars, 20 μm

**Figure 4:**
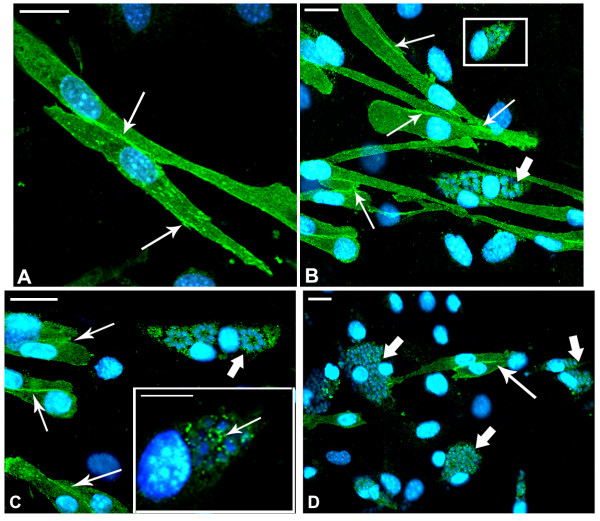
**Cadherin distribution in SkMC after 24 h of *T. gondii *interaction**. Confocal Microscopy analysis showing: (A) In 3-day-old SkMC cultures, after differentiation, myoblasts present intense cadherin labeling at the contact points (arrows). (B and C) In myoblasts after 24 h of interaction with *T. gondii *(thick arrow), cadherin (thin arrow) becomes disorganized forming aggregates at different sites, around and inside the parasitophorous vacuole (for detail, see inset). (D) Infected myoblasts after 24 h of interaction with *T. gondii *have little or no labeling for cadherin at points of cell-cell contact (thick arrow). Note that only uninfected cells show strong cadherin expression (thin arrow). Nuclei of cells and parasites labeled with DAPI, in blue. Bars, 20 μm

Indirect immunofluorescence assays were performed in order to localize cadherin, an adhesion molecule involved in homophilic recognition during myoblast and myotube fusion. In SkMC 2-day-old cultures, the myoblasts are still in multiplication and differentiation process. Cadherin is strongly revealed in every cell with higher fluorescence intensity in edges near the membrane and at the point of cell-cell contact (Figure 3A). Apparently, the existence of a single, newly internalized parasite did not lead to any change in the profile of cadherin distribution in host cells (Figure 3B), as demonstrated by immunofluorescence microscopy. The same results were maintained during the first 3 h of interaction (data not shown). After differentiation, myoblasts revealed cadherin highly concentrated at the cell-cell contact point (Figure 4A). However, this profile was not observed after 24 h of *T. gondii *infection. Besides disorganization, cadherin appeared in aggregates at different points of the SkMC, including around and inside the parasitophorous vacuole (Figure 4B and 4C - inset). Infected myoblasts showed low or no labeling for cadherin at cell-cell contact point (Figure 4B and inset and C). Even in cultures infected for 36 h, only uninfected cells present strong cadherin expression (Figure 4D).

**Figure 5:**
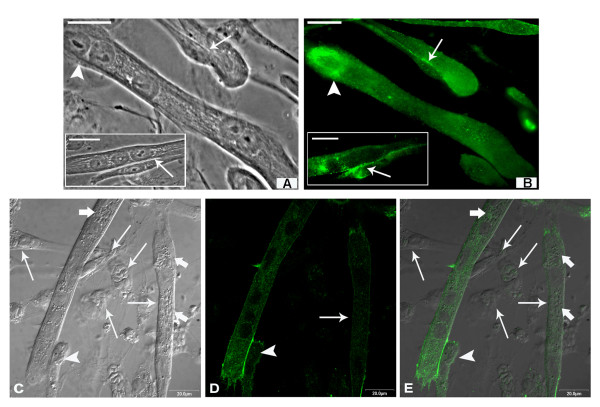
**Cadherin profile in differentiated cultures after 24 h of *T. gondii *interaction**. (A and inset) Mature (arrowhead) and young myotubes in fusion process with myoblasts (arrows) can be observed by phase contrast microscopy. (B and inset) By fluorescence microscopy, cadherin (in green) appears distributed throughout the myotubes, being more concentrated at the cell membrane during adhesion, while mature myotubes alone show more intense labeling at the extremities. (C) Interferential microscopy shows the adhesion of uninfected myoblasts (arrowhead) with a mature infected myotube (thick arrows). (D) Confocal microscopy analysis shows that infected myoblasts do not reveal cadherin labeling and more infected myotubes present weaker cadherin labeling (arrow). Observe that despite the weak labeling, in infected myotubes cadherin molecules appear to migrate to the point of contact with uninfected myoblasts (arrowhead). (E) Merge. Bars, 20 μm

During myogenesis *in vitro*, myoblasts interact with the surface of myotubes. The dynamics of this interaction induces the translocation of cadherin from the extremities of myotubes to the point of cell-cell contact (Figure 5A, B and inset). Labeling for cadherin was observed at the end of infected myotubes, especially at points of contact with uninfected myoblasts, suggesting migration of cadherin to the sites of possible membrane fusion (Figure 5C-E).

### Western blot analysis of cadherin expression in SKMC infected with *T. gondii*

**Figure 6:**
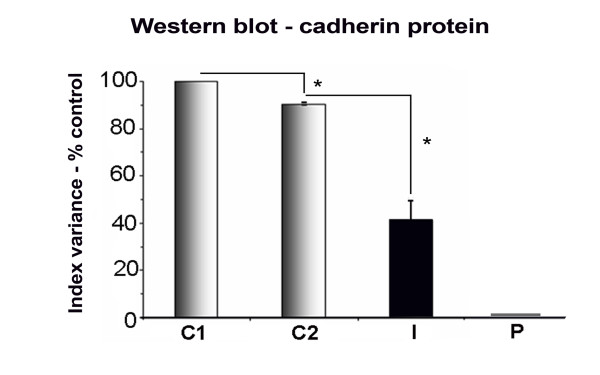
**Western blot analysis of cadherin protein expression**. (A) Percent index variance analysis of the western blot showing cadherin expression: (C1) 2-day old uninfected cultures, (C2) 3-day old uninfected SkMC (control), (I) cultures after 24 h of interaction with *T. gondii *tachyzoites, and (P) parasites alone (confirming the absence of synthesis of cadherin by *T. gondii *tachyzoites). Quantitative analysis revealed only 10% reduction in the expression of cadherin between normal cultures, reaching values of more than 50% reduction in *T. gondii *infected SkMC after 24 h. Results are representative of three independent experiments. Student's T-test (*) p ≤ 0.05.

The total cadherin pool was detected using a pan-cadherin-specific antibody, which recognizes the 130 kDa protein [27], since proteins were extracted from 2-3-day-old uninfected cultures (controls) and *T. gondii *24 h infected cultures. Quantitative data obtained by densitometric analysis showed that 3-day-old SkMC presented a reduction of only 10% in the synthesis of cadherin when compared to 2-day-old cultures. Regarding the participation of *Toxoplasma *in the modulation of cadherin synthesis, our data showed a significant decline of cadherin expression after 24 h of *T. gondii*-SkMC interaction, reaching a 54% reduction. These data demonstrate the variable rate of changes between infected and control SkMC during the analyzed period (Figure 6). For quantitative analysis, western blot signals were normalized against total proteins detected per lane in the corresponding MemCode stained membrane using the QuantityOne software (not shown).

### RT-PCR analysis of M-cadherin mRNA in SkMC- *T. gondii *infected cells

**Figure 7:**
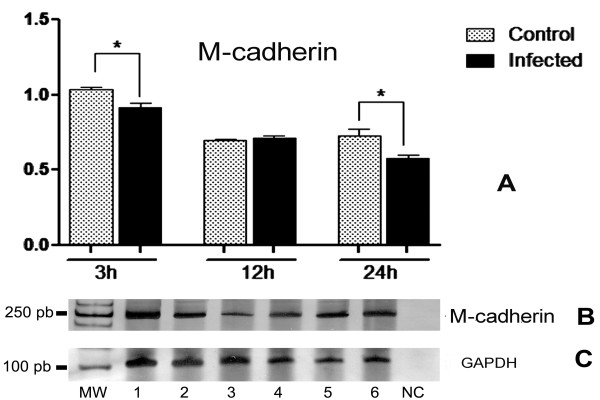
**Profile of M-cadherin mRNA expression by SkMC experimentally infected with *T. gondii***. (A) The arbitrary values presented in the graph are based on the densytometric analysis of the PCR gel image shown in panel B, corresponding to 3, 12 and 24 h of infection. Light bars indicate uninfected control cells and black bars indicate the infected cells. (B) Polyacrylamide, silver stained gels for visualization of the amplified M-cadherin and GAPDH mRNAs (from top to bottom, respectively). Lanes 1, 3 and 5 show the profiles of negative controls and lanes 2, 4 and 6 the profiles of infected cells (3, 12 and 24 h, respectively). NC, negative PCR control. Molecular size markers are indicated to the left. Student's T-test (*) p ≤ 0.05.

M-cadherin gene expression in SkMC experimentally infected with *T. gondii *was analyzed by RT-PCR. M-cadherin mRNA was detected 2 and 3 days after plating and it was up regulated only after the induction of myotube formation, which corresponds to the second day of culture. After 3 h of infection with *T. gondii *M-cadherin mRNA levels were significantly reduced and after 12 h of interaction, no change in M-cadherin mRNA expression profile was observed. However, after 24 h, M-cadherin mRNA expression was down regulated when compared to the corresponding SkMC control from 3 day-old cell cultures (Figure 7A-C).

## Discussion

This study analyzes the impact of *T. gondii-*infection on the myogenesis process. The results obtained showed that: (i) myoblasts are more susceptible to infection than myotubes; (ii) *T. gondii*-infected myoblasts are unable to fuse with others myoblasts and myotubes and, (iii) M-cadherin expression is down regulated during infection, indicating that *T. gondii *interferes with myogenesis in SkMC model.

We have observed that after 24 h of *T. gondii-*SkMC interaction, myoblasts are more infected than myotubes. This difference in infection levels possibly reflects the participation of cell surface molecules from both the parasite and host cells, acting as receptors/ligands, such as intercellular adhesion proteins with Ig domains (I-CAM, N-CAM and V-CAM) [36,37]. During infection and transmigration, *T. gondii *interacts with IgCAMs through the adhesion protein MIC2 released from micronemes, suggesting that the parasite infectivity capacity is at least partially dependent on the I-CAM molecules present on the host cell surface [38]. It has been established that during *in vivo *SkMC differentiation, a change in expression profile of adhesion molecules occurs: N-CAM and V-CAM, as well as cadherins, which are found in higher concentration in myoblasts than myotubes and in adult muscular fibers [27,29,39-44]. These data suggest that the different susceptibility of SkMC myoblasts and myotubes to infection by *T. gondii *tachyzoites can be related to the remodeling of adhesion molecule expression profiles on host cell surfaces during their differentiation.

The reproduction of the myogenesis process from mammalian embryonic skeletal muscle cells was demonstrated, as previously reported in both *in vivo *and *in vitro *studies [45-47]. It is well known that cadherin plays important roles in morphogenesis, such as cell recognition and cell rearrangement including myogenesis, both in the embryo and in the adult organism during regeneration [20,43,48]. Our results corroborated previous findings demonstrating that antibodies against cadherin protein recognize the same 130 kDa protein [27]. The 10% reduction observed in the synthesis of cadherin in 2- and 3 day-old cultures can be justified since, after 2 days of plating, some myoblasts have completed their proliferation and recognition programs [26]. In this manner, the infection carried out in cultures after 2 days of plating allowed the study of the role of *Toxoplasma *in cadherin modulation and inhibition of myogenesis.

We also demonstrated, by immunofluorescence, the distribution of cadherin throughout the myoblast surface, being more concentrated in aligned myoblasts and strongly localized at the point of cell-cell contacts. In young and mature myotubes, cadherin molecules were labeled on the sarcolemma and specifically accumulated at the extremities and on insertion sites of secondary myotubes [27,29,41-44]. In all SkMC (myoblasts and myotubes), no change was observed with respect to the cadherin distribution pattern during the first 3 h of interaction with *T. gondii*. However, infection of SkMC with *T. gondii *for more than 24 h resulted in the disruption of cadherin mediated cell junction with a sharp decline in the total cadherin pool. Our results showing, by confocal microscopy, the presence of cadherin around and inside the parasitophorous vacuole, open new perspectives to study the involvement of this adhesion protein during the interaction of *T. gondii *and muscle cells and also other cellular types not involved with the chronic phase of the disease.

In agreement with our immunofluorescence results, western blot analysis of cadherin expression showed no alteration in protein levels on newly infected myoblasts and myotubes (not shown). Nevertheless, a decrease in protein levels was observed after 24 h of interaction with *T. gondii*, which could lead to membrane fusion inhibition, interfering with the recognition process and fusion of myoblasts. Cultures analyzed after 24 h of *T. gondii *interaction, showed that the parasite can induce a reduction of more than 50% in cadherin protein expression, thus interfering with the myogenesis process.

Regarding the negative modulation of cadherin protein expression after 24 h of *T. gondii*-SkMC interaction, observed by western blot analysis, one factor that must be considered is the activation of proteolytic systems. It is known that, during the *T. gondii *lytic cycle proteolytic systems can be activated by molecules involved in the fusion process, including calcium ions (Ca^2+^) [49,50]. Previous works showed that, in response to the cytoplasmic Ca^2+ ^increase in *T. gondii *infected cells, there is an up-regulation of calpain activity which is involved in many biological events, including cell migration and muscle cell differentiation [51-54]. Thus, we suggest that in SkMC infected by *T. gondii *tachyzoite forms, the reduction observed in the cadherin expression profile may be, among other factors, due to modulation by Ca^2+ ^levels leading to an increase of calpain-3 proteolytic activity [48,54,55]. We believe that *T. gondii*, like other pathogens, can benefit from the modulation of cadherin and other adhesion molecules in order to facilitate migration to other neighboring cells and tissue.

Intracellular pathogens, such as *Helicobacter pylori, Shigella flexneri, Salmonella typhimurium, Trypanosoma cruzi *and *Chlamydia trachomatis *may module the adhesion junction molecules, such as E-cadherin, claudin-1, ZO-1, N-cadherin and nectin-1 affecting the adherent junctions [21,23,24,56-61]. However, this is not always a consistent behavior. For example, it was observed that in *Trichinella pseudospiralis *infected satellite cells from muscle cells, M-cadherin was up regulated; the same was not observed for *T. spiralis*, and the authors suggested a differential M-cadherin role in the infection process by different pathogens [25]. Similar to our immunofluorescence results, other authors have observed low or no staining for Pan- and N-cadherin in cardiomyocytes highly infected with *T. cruzi *leading to disruption of cadherin-mediated adheren junctions [24]. In our study, *T. gondii *infected SkMC after 3 and 24 h of interaction showed a significant reduction in cadherin mRNA levels, suggesting that *T. gondii *could be involved in the modulation of M-cadherin gene transcription. It has recently been described that *T. gondii *manipulates host signaling pathways, deploying parasite kinases and phosphatases and alters host cell gene transcription through rhoptry proteins [62,63]. An example is ROP16 that manipulates the host cell transcription factors STAT3 and STAT6 in the early infection. The rhoptry proteins may alter host cell gene transcription and set up an environment that favors *Toxoplasma *replication and survival. Another example is the inhibition of STAT1 during *T. gondii *interaction, which possibly increases its pathogenicity [62-64].

During embryonic development the formation and maintenance of muscle tissues primarily requires the action of adhesion proteins such as cadherins [43]. In our *in vitro *studies using SkMC we verified that *T. gondii *affected the myogenesis process by negatively regulating cadherin expression. Thus, we believe that our results can contribute to a further investigation of congenital infection by *Toxoplasma *during the embryonic formation of muscle tissue.

## Conclusions

The data of this paper reveal that during the interaction between *T. gondii *tachyzoite forms and primary culture of SkMC, myoblasts are more susceptible to infection than myotubes. These data suggest that the different susceptibility of SkMC myoblasts and myotubes to infection by *T. gondii *can be related: (i) to the remodeling of the host cell's surface adhesion molecule expression profiles during their differentiation; (ii) to the participation of cell surface molecules from both parasite and host cells, acting as receptors/ligands, such as N-CAM and V-CAM, as well cadherins, which are found in higher concentration in myoblasts than myotubes and in adult muscular fibers [27,29,39-42]. We also demonstrated that *T. gondii *SkMC infection down regulates M-cadherin mRNA expression, leading to molecular modifications in the host cell surface which disarray the contact sites between myoblasts and myoblasts-myotubes, promoting the instability of the junctions, which interferes with membrane fusion and consequently inhibiting the myogenesis process. These changes, could lead to the modulation of other molecules contributing to toxoplasmosis pathogenesis in the muscle tissue.

## Competing interests

The authors declare that they have no competing interests.

## Authors' contributions

HSB conceived, participated in the design and coordination of the study and had the general supervision and complete overview of the project. AFG co-conceived the study, carried out most of the experimental work, including the processing of samples and the final illustrations for the manuscript, analyzed data and drafted the manuscript, as part of her PhD thesis. EVG and LC participated in the design of the study. JRC performed western blot analysis. LML carried out the molecular assays. All authors analyzed the data and read and approved the final manuscript.
